# Supplementary material for: Analysis of Fluid Balance and Urine Values in Elite Soccer Players: Impact of Different Environments, Playing Positions, Sexes, and Competitive Levels
Source: Nutrients. 2024 Mar 21;16(6):903. doi: 10.3390/nu16060903 (PMC10975990; doi:10.3390/nu16060903)
Supplement: Supplementary file 1 [file nutrients-16-00903-s001.zip › nutrients-2895457-supplementary.pdf]

Table S1. Hydration values and fluid balance of the sample as a function of playing position.

RM sample

		Goalkeeper (n=4)	Defender (n=7)	Midfielder (n=8)	Forward (n=8)	Total (n=27)
W	Weight loss:					
	kg	0.52 (0.46)	0.56 (0.51)	1.07 (0.68)†	0.43 (0.50)	0.67 (0.59)*
	%	0.69 (0.59)	0.79 (0.77)	1.56 (0.90)†	0.58 (0.71)	0.94 (0.84)
	Fluid intake (l)	1.18 (0.38)	1.51 (0.83)	1.96 (0.82)†	1.68 (0.52)†	1.64 (0.70)*
	Urine output (l)	0.14 (0.07)	0.21 (0.15)	0.24 (0.16)	0.21 (0.13)	0.20 (0.13)†
	SR:					
	l/h	0.78 (0.23)	0.94 (0.28)†	1.39 (0.55)†	0.96 (0.30)†	1.05 (0.43)*
	ml/min	13.02 (3.83)	15.58 (4.71)†	23.24 (9.21)†	15.94 (4.95)†	17.58 (7.16)*
	USG	1.02 (0.01)	1.02 (0.01)	1.02 (0.01)	1.02 (0.01)	1.02 (0.01)*
C	Weight loss:					
	kg	0.34 (0.25)	0.52 (0.44)	0.31 (0.34)	0.22 (0.40)	0.34 (0.37)
	%	0.48 (0.36)	0.71 (0.56)	0.45 (0.45)	0.32 (0.61)	0.48 (0.52)
	Fluid intake (l)	0.90 (0.24)	0.97 (0.48)	0.84 (0.40)	1.06 (0.38)	0.95 (0.38)
	Urine output (l)	0.11 (0.04)	0.16 (0.12)	0.11 (0.06)	0.18 (0.08)	0.11 (0.08)
	SR:					
	l/h	0.57 (0.22)	0.67 (0.12)	0.52 (0.16)	0.55 (0.07)	0.57 (0.14)
	ml/min	9.44 (3.72)	11.10 (1.93)	8.66 (2.61)	9.19 (1.18)	9.56 (9.67)
	USG	1.02 (0.01)	1.02 (0.01)	1.02 (0.01)	1.03 (0.01)	1.02 (0.01)

n-RM sample

	Goalkeeper			Defender			Midfielder			Forward			Total		
	W (n=5)	C (n=3)	T (n=8)	W (n=24)	C (n=8)	T (n=32)	W (n=19)	C (n=12)	T (n=31)	W (n=14)	C (n=2)	T (n=16)	W (n=62)	C (n=25)	T (n=87)
Weight loss:															
kg	0.50 (0.40)	0.33 (1.28)	0.44 (0.75)	0.67 (0.59)	0.34 (0.24)	0.59 (0.54)	0.72 (0.63)	0.39 (0.38)	0.59 (0.56)	0.48 (0.54)	0.50 (0.07)	0.48 (0.50)	0.63 (0.57)	0.37 (0.47)	0.55 (0.56)

%	0.69 (0.51)	0.41 (1.54)	0.58 (0.91)	0.98 (0.79)	0.47 (0.35)	0.85 (0.73)	1.06 (0.89)	0.55 (0.56)	0.86 (0.81)	0.66 (0.73)	0.84 (0.15)	0.68 (0.68)	0.91 (0.79)	0.53 (0.62)	0.80 (0.76)†
Fluid intake (l)	1.25 (0.36)	1.36 (0.92)	1.29 (0.57)	1.64 (0.67)	0.86 (0.30)	1.44 (0.69)†	1.89 (0.73)	0.92 (0.45)	1.51 (0.79)†	1.59 (0.52)	0.90 (0.85)	1.50 (0.58)	1.67 (0.65)	0.95 (0.50)	1.46 (0.69)†
Urine output (l)	0.14 (0.06)	0.15 (0.11)	0.14 (0.07)	0.19 (0.13)	0.20 (0.14)	0.19 (0.13)	0.18 (0.13)	0.15 (0.07)	0.17 (0.11)	0.18 (0.11)	0.05 (0.01)	0.16 (0.11)	0.18 (0.12)	1.70 (0.10)	0.17 (0.12)
SR:															
l/h	0.80 (0.20)	0.77 (0.24)	0.79 (0.20)	1.06 (0.45)	0.50 (0.15)	0.92 (0.46) †	1.21 (0.46)	0.58 (0.13)	0.97 (0.48)†	0.95 (0.33)	0.68 (0.39)	0.91 (0.34)	1.06 (0.43)	0.58 (0.18)	0.92 (0.43)†
ml/min	13.37 (3.41)	12.89 (4.0)	13.19 (3.35)	17.63 (7.51)	8.31 (2.53)	15.30 (7.75) †	20.20 (7.74)	9.63 (2.1)	16.11 (8.06)†	15.76 (5.50)	11.29 (6.42)	15.20 (5.59)	17.65 (7.10)	9.73 (3.00)	15.38 (7.16)†
USG	1.02 (0.01)	1.03 (0.01)	1.02 (0.00)	1.02 (0.01)	1.03 (0.01)	1.02 (0.01)	1.02 (0.01)	1.02 (0.01)	1.02 (0.01)	1.02 (0.01)	1.03 (0.00)	1.02 (0.01)	1.02 (0.01)	1.03 (0.01)	1.02 (0.01)

W: Summer measurement; C: Winter measurement; SR: Sweat rate; T: Total; USG: Urine specific gravity. Variables are expressed as mean (SD). *p<0.05 playing position. †p<0.05 warm vs cool.

Table S2. Hydration values and fluid balance of the sample as a function of playing position.

RM sample. (n (%))

		Goalkeeper (n=4)		Defender (n=7)		Midfielder (n=8)		Forward (n=8)		Total (n=27)	
		CO	USG	CO	USG	CO	USG	CO	USG	CO	USG
H:	*W*	0 (0.0)	1 (25.0)	1 (14.3)	2 (28.6)	1 (12.5)	3 (37.5)	1 (12.5)	2 (25.0)	3 (11.1)	8 (29.6)
	C	0 (0.0)	2 (50.0)	0 (0.0)	2 (28.6)	0 (0.0)	2 (25.0)	0 (0.0)	1 (12.5)	0 (0.0)	7 (25.6)
DH:	*W*	3 (75.0)	0 (0.0)	4 (57.1)	2 (28.6)	6 (75.0)	3 (37.5)	5 (62.5)	2 (25.0)	18 (66.7)	7 (25.9)
	C	4 (100.0)	0 (0.0)	7 (100.0)	0 (0.0)	8 (100.0)	2 (25.0)	8 (100.0)	3 (37.5)	27 (100.0)	5 (18.5)
SDH:	*W*	1 (25.0)	3 (75.0)	2 (28.6)	3 (42.9)	1 (12.5)	2 (25.0)	2 (25.0)	4 (50.0)	6 (22.2)	12 (44.4)
	C	0 (0.0)	2 (50.0)	0 (0.0)	5 (71.4)	0 (0.0)	4 (50.0)	0 (0.0)	4 (50.0)	0 (0.0)	15 (55.6)

n-RM sample. (n (%))

	Goalkeeper		Defender		Midfielder		Forward		Total	
	CO	USG	CO	USG	CO	USG	CO	USG	CO	USG

H:	*W*	0 (0.0)	2 (40.0)	4 (16.7)	6 (25.0)	2 (10.5)	6 (31.6)	1 (7.1)	2 (14.3)	7 (11.3)	16 (25.8)
	C	0 (0.0)	1 (33.3)	0 (0.0)	3 (37.5)	0 (0.0)	2 (16.7)	0 (0.0)	0 (0.0)	0 (0.0)	6 (24.0)
DH:	*W*	4 (80.0)	0 (0.0)	17 (70.8)	8 (33.3)	16 (84.2)	7 (36.8)	9 (64.3)	6 (42.8)	46 (74.2)	21 (33.9)
	C	3 (100.0)	0 (0.0)	7 (87.5)	1 (12.5)	12 (100.0)	4 (33.3)	1 (50.0)	0 (0.0)	[illegible]3 (92.0)	5 (20.0)
SDH	*W*	1 (20.0)	3 (60.0)	3 (12.5)	10 (41.7)	1 (5.3)	6 (31.6)	4 (28.6)	6 (42.8)	9 (14.5)	25 (40.3)
:	*C*	0 (0.0)	2 (66.7)	1 (12.5)	4 (50.0)	0 (0.0)	6 (50.0)	1 (50.0)	2 (100.0)	2 (8.0)	14 (56.0)

W: Summer measurement; C: Winter measurement; CO: Urine colour; DH: Dehydrated; H: Hydrated; SDH: Seriously dehydrated; USG: Urine specific gravity. Variables are expressed as n (%). *p<0.05 hydration status vs playing position.

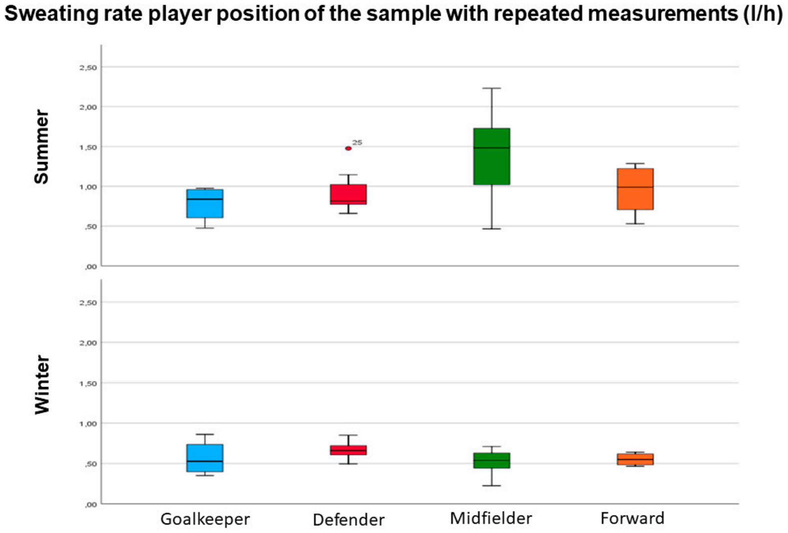

Figure S1. Sweating rate by player position of the sample with repeated measurements.

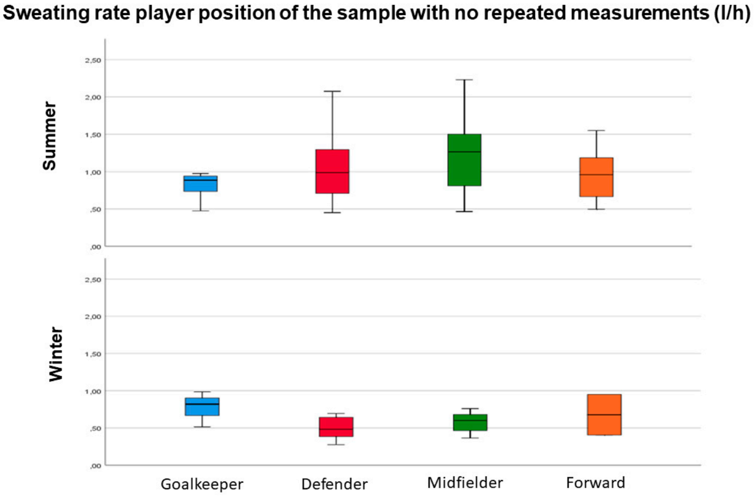

Figure S2. Sweating rate by player position of the sample with non-repeated measurements.

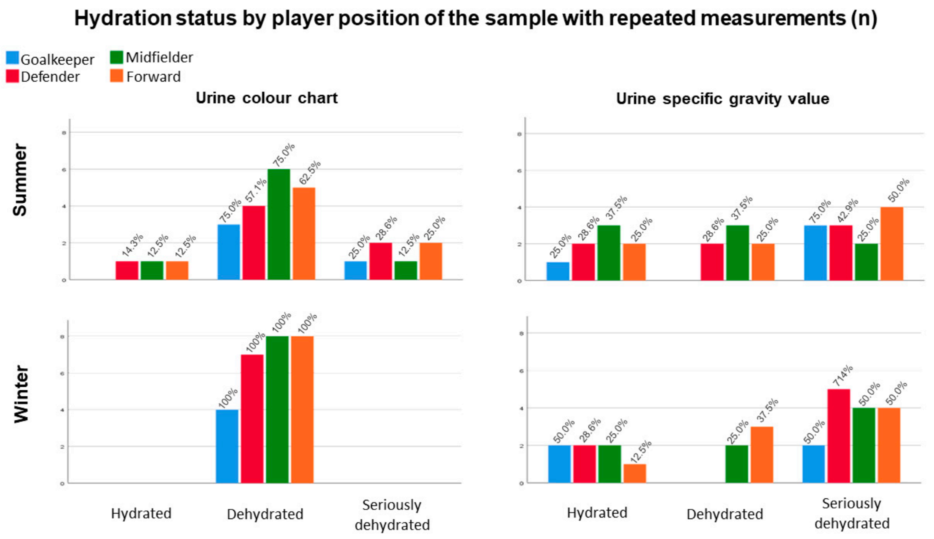

Figure S3. Hydration status by player position of the sample with repeated measurements

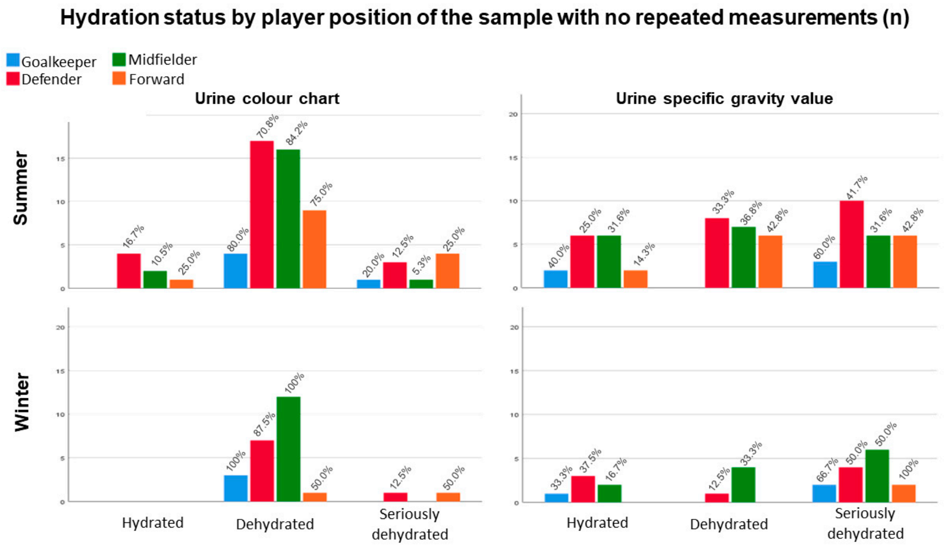

Figure S4. Hydration status by player position of the sample with non-repeated measurements.
